# The Impact of Over-The-Counter Lactic Acid Containing Vaginal Gels on the Integrity and Inflammatory State of the Vaginal Epithelium *in vitro*

**DOI:** 10.3389/frph.2022.915948

**Published:** 2022-06-23

**Authors:** David Tyssen, Anna C. Hearps, Kalyani Guntur, Lindi Masson, Simon Cook, Simon E. Moulton, Jacques Ravel, Catriona S. Bradshaw, Seyoum Ayehunie, Gilda Tachedjian

**Affiliations:** ^1^Life Sciences Discipline, Burnet Institute, Melbourne, VIC, Australia; ^2^Central Clinical School, Monash University, Melbourne, VIC, Australia; ^3^MatTek Corporation, Ashland, MA, United States; ^4^Department of Pathology, Institute of Infectious Disease and Molecular Medicine, University of Cape Town, Cape Town, South Africa; ^5^Centre for the AIDS Programme of Research in South Africa, Durban, South Africa; ^6^Australian Research Council (ARC) Centre of Excellence for Electromaterials Science, School of Science, Computing and Engineering Technologies, Swinburne University of Technology, Melbourne, VIC, Australia; ^7^Aikenhead Centre for Medical Discovery, St. Vincent's Hospital Melbourne, Melbourne, VIC, Australia; ^8^Iverson Health Innovation Research Institute, Swinburne University of Technology, Melbourne, VIC, Australia; ^9^Institute for Genome Sciences, University of Maryland School of Medicine, Baltimore, MD, United States; ^10^Department of Microbiology and Immunology, University of Maryland, Baltimore, MD, United States; ^11^Melbourne Sexual Health Centre, Alfred Health, Melbourne, VIC, Australia; ^12^Melbourne School of Population and Global Health, The University of Melbourne, Melbourne, VIC, Australia; ^13^Department of Microbiology, Monash University, Clayton, VIC, Australia; ^14^Department of Microbiology and Immunology at the Peter Doherty Institute for Infection and Immunity, University of Melbourne, Melbourne, VIC, Australia

**Keywords:** lactobacilli, lactic acid, vaginal microbiome, vaginal gel, cervicovaginal epithelium

## Abstract

The vaginal microbiome influences a wide range of health outcomes in women, where a microbiome dominated by *Lactobacillus* spp. is considered optimal and associated with reduced risk of pre-term birth and acquisition of sexually transmitted infections including HIV. Conversely, replacement of lactobacilli by non-optimal bacteria leads to the development of bacterial vaginosis, which is associated with increased risk of these outcomes. Lactobacilli produce the metabolite lactic acid (LA) which is a potent antibacterial and antiviral agent. The potential therapeutic benefits of LA have prompted the development of numerous over-the-counter LA-containing gels for use in the vagina, although a comprehensive analysis of the impact of these formulations on the cervicovaginal epithelium and pro-inflammatory cytokine/chemokine responses, has not been assessed. Here, we evaluated the properties of 11 over-the-counter gels, including 9 containing LA, marketed for use in the vagina. Ten of the 11 gels had an osmolality greater than vaginal fluid from women with *Lactobacillus-*dominated microbiota (370 ± 40 mOsmol/kg in women with Nugent score 0–3), with six gels that were hyperosmolal >2,000 mOsmol/kg. Using a reconstructed primary cell model of the vaginal epithelium, we found hyperosmolal gels had a detrimental impact on epithelial barrier integrity, resulting in substantial cellular toxicity (<10% viability as compared to untreated cells) and reduced epithelial barrier integrity [≈30% of untreated cells, assessed by transepithelial electrical resistance (TEER)]. Treatment of vaginal tissues with most of the gels elicited the production of pro-inflammatory factors including IL-1α (8 of 11) and IL-1β (10 of 11) which are associated with heightened risk of HIV acquisition *in vivo*. The majority of the OTC gels elicited moderate tissue damage as determined by histology. The detrimental effects of these gels on the human vaginal epithelium *in vitro* may predict compromised epithelial barrier integrity and genital inflammation *in vivo*, which has implications for sexual and reproductive health. This study highlights the importance of evaluating the impact of intravaginal products on the integrity and inflammatory status of the mucosal epithelium to avoid unfavorable off target effects.

## Introduction

The cervicovaginal microbiome has a substantial influence on sexual and reproductive health outcomes. Women colonized with an “optimal” vaginal microbiota dominated by beneficial *Lactobacillus* spp. have a vaginal pH <4.5 and experience a lower risk of sexually transmitted infections (STIs) including HIV, and adverse birth outcomes (1–4). In contrast, bacterial vaginosis (BV), which is the most common clinical manifestation of a non-optimal vaginal microbiota in women of reproductive age, is characterized by an increase in the load of anaerobic bacteria and a decrease in the relative abundance of lactobacilli, a vaginal pH >4.5, and is associated with an increased risk of acquiring HIV and other STIs as well as adverse birth outcomes (5).

Vaginal lactobacilli produce the metabolite lactic acid (LA), which is a bioactive that acidifies the vagina to a pH ranging from 3.2 to 4.2 (6). *In vitro*, lactobacilli growth is dependent on pH, with bacterial growth and media acidification slowing as an asymptotic pH of 3.2–4.8 is reached (7). The levels of LA in women with an optimal vaginal microbiome is on average ~1% (6). Vaginal LA comprises different ratios of L- and D- isomers (8) that are predominately produced by vaginal bacteria, with *L. crispatus* typically producing more D- vs. L-LA (9). At pH <3.86, LA is predominately in the uncharged biologically active form, while at pH >3.86 it is mainly found as the inactive lactate anion.

Physiological levels of LA in women with an optimal vaginal microbiome have virucidal activity against HIV and herpes simplex virus (HSV) *in vitro* (10–12). LA also inhibits the growth of bacterial STIs including *Chlamydia trachomatis* (13) and *Neisseria gonorrhoeae* (14), and kills 17 different bacteria associated with BV, but not vaginal lactobacilli (15). Treating vaginal epithelial cells with D-LA prevents chlamydia infection (16), while the presence of this isomer is associated with trapping HIV in vaginal mucous (17). Thus, LA can potentially prevent STIs by distinct mechanisms including direct inactivation of the pathogen or by acting directly on the cervicovaginal epithelium to prevent bacterial infections.

In addition to its potent virucidal and bactericidal activity, LA induces an anti-inflammatory state in human cervicovaginal epithelial cells and inhibits microbe- or pathogen- associated molecular pattern-stimulated production of proinflammatory cytokines and chemokines (18, 19). This is consistent with the observed *in vivo* association between a *Lactobacillus*-dominated microbiome and a low/non-inflammatory vaginal environment, in contrast to women with BV who exhibit high cervicovaginal levels of soluble pro-inflammatory factors (20–22). Heightened vaginal inflammation is associated with increased risk of HIV acquisition mediated through pro-inflammatory immune mediators that recruit HIV-target cells to the superficial layers of the epithelium (2, 21, 23), as well as by disrupting cervicovaginal epithelial barrier integrity (24).

In addition to immunological defenses, the cervicovaginal epithelium is an important physical barrier to pathogen invasion. Proteomic analyses of cervicovaginal secretions have identified an association between *Lactobacillus*-dominated microbiota and a signature of enhanced epithelial barrier integrity (25, 26) whilst lactobacilli enhance epithelial wound healing *in vitro* (27). We have recently identified LA directly upregulates expression of epithelial barrier-related genes and enhances cervicovaginal epithelial barrier function (28).

Accordingly, there is an increasing body of evidence to support the notion that administration of LA *in vivo* could mitigate genital inflammation associated with conditions such as BV, enhance epithelial barrier integrity and may support a shift from a non-optimal toward a *Lactobacillus*-dominated optimal microbiota to facilitate improved genital and reproductive health. These potential therapeutic benefits of LA have been recognized, and numerous over- the-counter (OTC) vaginal gels and treatments containing LA are available for indications including treating BV, vaginal irritation and as part of a non-hormonal contraceptive for preventing unplanned pregnancies (29). However, high quality evidence to support their clinical efficacy is scant (29) and their impact on the cervicovaginal epithelium remains to be fully defined. We therefore evaluated the impact of OTC vaginal gels on the viability, integrity and inflammatory status of the cervicovaginal epithelium to inform the development of efficacious LA-containing vaginal gel formulations with an optimal safety profile.

## Materials and Methods

### LA Content and Biochemical Properties of Gels

OTC gels analyzed in this study are listed in Table 1. The osmolality of gels was measured in triplicate using a cryoscopic osmometer (Gonotec Osmomat 030-D, Gemini BV, Apeldoorn, Netherlands) calibrated at 100, 300, and 2,000 mOsmol/kg. Gels which initially gave values above the upper limit of quantitation (3,000 mOsmol/kg) were diluted 1:2 in H_2_O and measured again, with the reassessed values expressed as an extrapolated value. Gel pH was measured using a pH electrode connected to a pH meter (TPS-Aqua meter and IJ sensor, Vintessential Laboratories, Orange, Australia) which allows accurate pH measurement of viscous fluids. LA levels were measured using the D-lactic acid/L-lactic acid kit (R-Biopharm, Darmstadt, Germany) as previously described (11). This method measures the concentration of D- and L-lactate anions after neutralization of the sample to pH 8–10 to calculate the total lactate concentration in the sample. The concentration of protonated LA present in undiluted gels was then calculated using the sample pH and the Henderson-Hasselbalch equation and a pKa value for LA of 3.86 as previously described (6).

**Table 1:** Physicochemical properties of over-the-counter (OTC) gels.

**Gel product**	**Manufacturer**	**Use**	**pH**	**Osmolality^a^ (mOsmol/kg)**	**Total LA** **(% w/v)**	**Ratio of D:L LA**
Caya Diaphragm Gel^b^	Delta Med GmbH, Germany	Lubricant/ Diaphragm gel	3.87	**1,614 ±10**	7.28 ± 0.05	0
ContraGel green^b^	Delta Med GmbH, Germany	Lubricant/ Diaphragm gel	3.89	**1,549 ±2**	7.12 ± 0.11	0
Lactigel	Laboratories Liconsa S.A, Spain	Vaginal pH	4.16	**>3,000 [5,002 ±52]**	5.18 ± 0.17	0.97
Relactagel	Kora Healthcare, Dublin, Ireland	BV	3.75	**>3,000 [3,770 ±70]**	5.01 ± 0.14	0.93
Geliofil Classic	Rolf Kullgren, Sweden	Vaginal irritation/BV	3.76	**>3,000 [3,392 ±38]**	5.01 ± 0.10	0
Balance Activ	Rolf Kullgren, Sweden	BV	3.78	**>3,000 [4,308 ±215]**	4.97 ± 0.09	0
Canesbalance	Rolf Kullgren, Sweden	BV	3.79	**>3,000 [3,576 ±8]**	4.84 ± 0.13	0
Gynofit	Tentan AG, Itingen, Switzerland	Vaginal infections including BV	3.76	**2026 ±6**	1.43 ± 0.04	0
Restore	Good Clean Love, Oregon, USA	Vaginal moisturizer/pH balance	3.73	293 ±0.3	1.06 ± 0.05	0.95
Aci-Jel Balance	Care Pharmaceuticals, Australia	Vaginal pH	4.11	**902 ±4**	0 ± 0	N/A
Fleurstat (1% astodrimer)	QualRep Services BV, Netherlands	BV	4.82	454 ±2	0 ± 0	N/A

*Osmolality and lactic acid (LA) data show mean and standard error the mean (SEM) from n = 3 replicates*.

a *Osmolality of healthy cervicovaginal fluids is 370 +/- 40 mOsmol/kg. Bold indicates hyperosmolal gels. Osmolality measurements in square brackets indicate extrapolated values*.

b *Based on packaging and biochemical properties, Caya gel and ContraGel are likely the same formulation*.

### Treatment of Organotypic EpiVaginal Tissue Model With Gels

The impact of OTC gels on the cervicovaginal epithelium was assessed using the EpiVaginal (VEC-100) reconstructed human 3-dimensional vaginal epithelial tissue model (MatTek Corp., Ashland, MA) by researchers who were blinded to the identification of the test articles. The MatTek model is composed of healthy primary vaginal epithelial cells in a highly differentiated structure consisting of basal, supra-basal and surface glycogen-containing epithelial cell layers that closely resemble the structure of human vaginal explants (30). The source of primary cervicovaginal epithelial cells for generation of the MatTek VEC-100 tissue was human vaginal-ectocervical tissue obtained from otherwise healthy women (age 29–48) undergoing hysterectomies for benign indications via the National Disease Research Interchange (NDRI, Philadelphia, PA) following Internal Review Board (IRB) approval. Neat gel (100 μL) was added apically to the EpiVaginal model and incubated at 37°C with 5% CO_2_ for 4 or 24 h_._ Gynol II (a spermicide containing 3% Nonoxynol-9) was used as a positive control as previously described (31) whilst hydroxyethyl cellulose (HEC) gel, which is an inert gel that has been used extensively in vaginal gels that have been evaluated *in vivo* (32–35) and designed to minimize negative effects on the vaginal mucosa, served as a placebo/negative control. Culture supernatant was collected from the basolateral chamber of treated tissues after 24 h and stored at −80°C for subsequent analysis. Tissue viability was determined using the MTT [3-(4,5-dimethylthiazol-2-yl)-2,5-diphenyltetrazolium bromide] assay (36). Epithelial barrier integrity was assessed by transepithelial electrical resistance (TEER) using an EVOM volt-ohmmeter equipped with an EndOhm electrode chamber (World Precision Instruments, Sarasota, FL) as previously described (31). TEER measurements were expressed relative to untreated tissue. TEER and viability were evaluated across two independent assays with three replicate tissues per condition per assay as described previously (30, 37). Average coefficients of variation for TEER and viability for all treatments/time points were 12.7 and 2.6%, respectively; full detail of individual results is shown in Supplementary Table 1. Gels were considered acceptable in this model if they demonstrated ≥50% viability and TEER at 24 h post treatment compared to untreated vaginal tissue.

### Assessment of Cytokine/Chemokine Production

Frozen culture supernatants (*n* = 3 per treatment) were thawed at room temperature and 50 μl of each sample was tested in duplicate. Macrophage inflammatory protein-3α (MIP3α), interleukin (IL)-1β, IL-6, IL-1α and IL-1 receptor antagonist (IL-1RA) were quantified using the Human Magnetic Luminex assay following manufacturer's protocol (R&D Systems, Minneapolis, MN). Luminex data was collected and analyzed on a BioPlex 200 multiple immunoassay system (Bio-Rad, Hercules, CA). IL-8 was measured in duplicate samples by ELISA (R&D Systems) after dilution of culture supernatants (1:50) in supplied diluent reagent following the manufacturer's protocol.

### Histological Assessment of Epithelium

After 24 h of treatment, tissue samples were fixed in 10% formalin (overnight at room temperature). Tissue cross-sections were cut (5–7 μm thick), mounted on microscopic slides, and stained with hematoxylin and eosin (H & E). A histopathogy-experienced technician assessed and graded each tissue section according to a number of parameters that were (a) loss of glycogen filled layers, (b) tissue thinning, (c) separation or perturbation of apical and glycogen filled layers, and (d) reorganization/disorganization of suprabasal, apical and glycogen layers. These four parameters, each carrying different weightings, were then used to determine an overall tissue damage score.

## Results

### Composition and pH of Vaginal Gels

Eleven OTC gels were analyzed including seven LA-containing gels for BV (Lactigel, Relactagel, Balance Activ, Gynofit, Canebalance, Geliofil, and Restore), two gels used as a non-hormonal contraceptive (Contragel and Caya Gel; likely to be the same formulation based on packaging and composition; Table 1) and two vaginal gels that do not contain LA (Aci-Jel and Fleurstat, where the active ingredients are acetic acid and astodrimer, respectively). All 11 gels had similar pH values of between 3.75 and 4.82. Nine of the 11 gels contained LA at levels ranging from 1.06 to 7.28% (w/v). Three of these gels were formulated with L- and D-LA (racemic) while the other LA-containing gels solely comprise the L-LA isomer (Table 1).

### The Majority of OTC Vaginal Gels Containing LA Were Hyperosmolal

The osmotic concentration (measured as osmolality) of cervicovaginal fluid from women with *Lactobacillus*-dominated vaginal microbiota (Nugent 0–3) is ~370 +/- 40 mOsmol/kg of fluid (31). However, the vast majority of vaginal lubricants and moisturizers are hyperosmolal (31, 38, 39) and are associated with mucosal irritation (40), decreased epithelial cell viability and barrier integrity *in vitro* (31, 39, 41), and cause rectal epithelial damage and denudation *in vivo* (42). For these reasons, the World Health Organization (WHO) recommends the osmolality of vaginal lubricants should ideally not exceed 380 mOsm/Kg, although given the limited availability of products meeting this criterion, an interim upper limit of 1,200 mOsm/Kg is recommended (43).

We measured the osmolality of 11 OTC gels recommended for vaginal use. Two gels were essentially iso-osmolal; Restore [Good Clean Love; previously evaluated in EpiVaginal tissue by Ayehunie et al. (31)] had an osmolality of 293 mOsmol/kg whilst that of Fleurstat was 454 mOsmol/kg. Another gel (Aci-jel Balance) was hyperosmolal (902 mOsmol/kg) but below the WHO recommendation of 1,200 mOsm/Kg. The remaining 8 gels evaluated were all hyperosmolal with many exhibiting an osmolality up to 10-fold greater than physiological levels (Table 1).

### Hyperosmolal Gels Had a Detrimental Effect on Epithelial Cell Viability and Barrier Integrity

**Figure 1:**
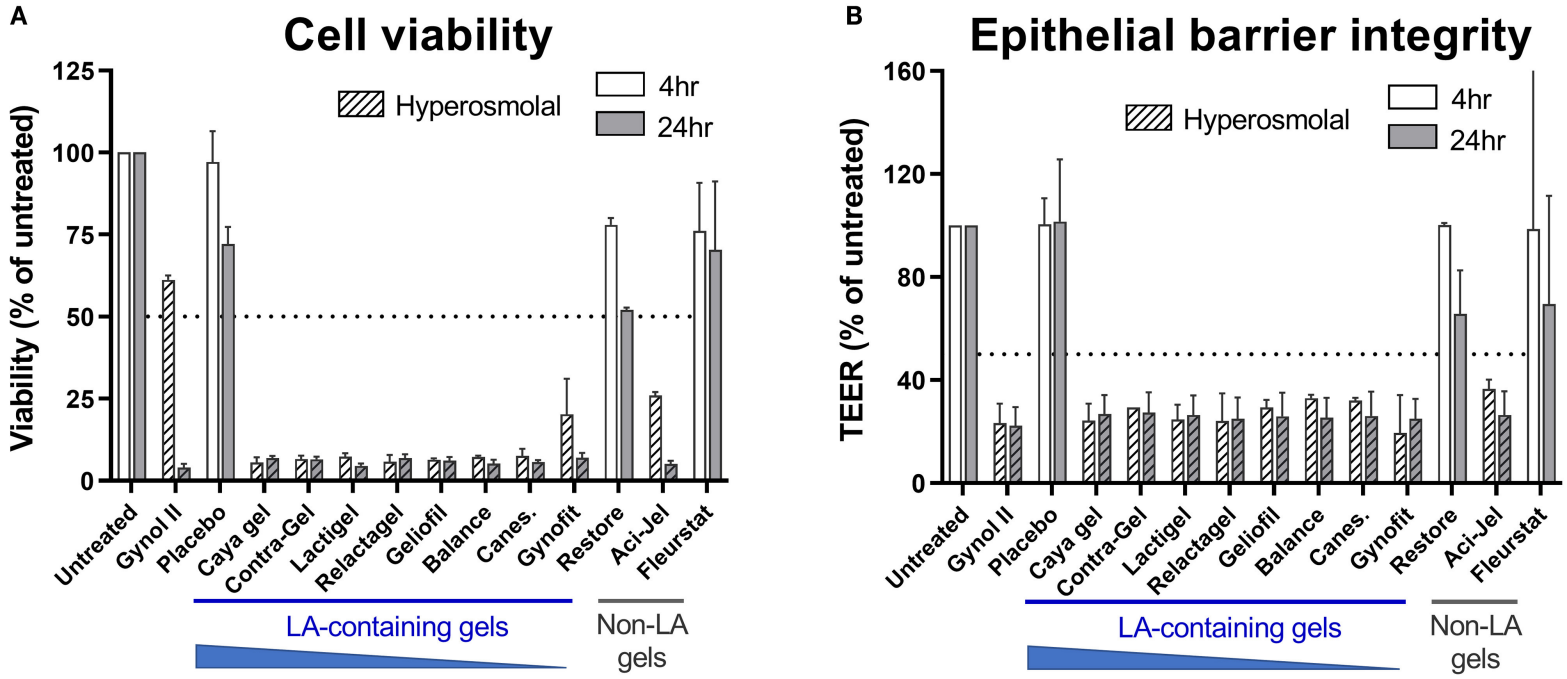
Effects of lactic acid (LA)-containing gels on vaginal epithelial cell viability and integrity. The EpiVaginal tissue model was treated with 11 over-the-counter (OTC) vaginal gels, the positive control gel Gynol II or a hydroxyethyl cellulose placebo gel as indicated. Cell viability **(A)** was assessed by MTT assay and epithelial barrier integrity **(B)** measured by transepithelial electrical resistance (TEER) at 4 or 24 h after gel addition. Hashed bars indicate hyperosmolal gels. A and B show mean and standard deviation of data derived from *n* = 2 independent experiments with 3 individual tissue replicates per treatment included in each assay. Values below 50% of untreated (indicated by dotted line in graphs) is considered a substantial effect.

To evaluate the impact of gel hyperosmolality on viability of the cervicovaginal epithelium we utilized the EpiVaginal human primary tissue model which recapitulates the multilayer morphology of the vaginal epithelium (30). Consistent with previous reports regarding the cytotoxicity of hyperosmolal gels (31), treatment of EpiVaginal tissues with most OTC vaginal gels resulted in substantial reduction in cell viability below the acceptable level (designated as <50% of the untreated sample) within 4 h of treatment, which was more rapid than the positive control gel containing Nonoxynol-9 where substantial cytotoxicity was only observed at 24 h (Figure 1A). Only two OTC gels (Fleurstat and Restore; the latter containing 1% LA) were found to be non-toxic, and these gels exhibited osmolalities similar to physiological levels.

Given the substantial effects of hyperosmolal gels on epithelial cell viability, we evaluated the barrier function of cervicovaginal cells after treatment. Consistent with the viability data, all hyperosmolal gels had a detrimental effect on epithelial barrier integrity as assessed by TEER, with barrier function reduced to ~30% compared to that of untreated cells within 4 h of treatment (Figure 1B). In contrast, barrier integrity was largely maintained following treatment with gels which were iso-osmolal (i.e., Restore and Fleurstat). Together, these data indicate the profound effect of hyperosmolal gels on the viability and barrier function of the cervicovaginal epithelium and reiterate the importance of osmolality in influencing intravaginal gel properties.

### OTC Vaginal Gels Elicit the Production of Inflammatory Cytokines From Cervicovaginal Epithelial Cells

**Figure 2:**
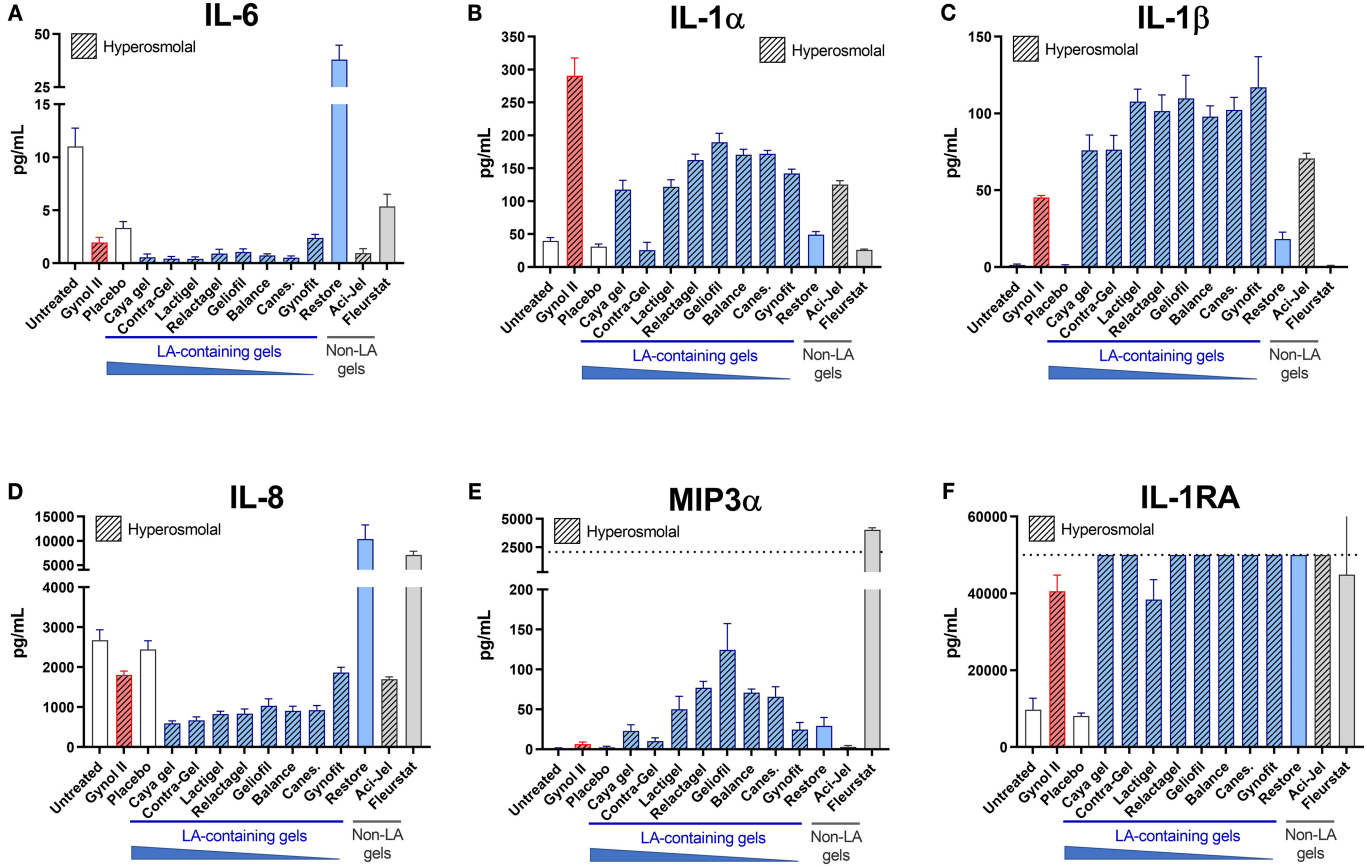
Effects of LA-containing gels on inflammatory mediator production. The effect of over-the-counter (OTC) vaginal gels on the production of inflammatory factors from the EpiVaginal tissue model was assessed after 24 h of treatment. Levels of IL-6 **(A)**, IL-1α **(B)**, IL-1β **(C)**, IL-8 **(D)**, MIP3α **(E)** and IL-1RA **(F)** were measured in cell culture supernatants of treated tissues by Luminex assay. Hashed bars indicate hyperosmolal gels. Dotted lines indicate upper limit of quantitation (LOQ) of the assays in panels E and F, respectively; all other analytes were within the assay range. Graphs show mean and SEM of *n* = 6 replicates from two independent assays.

Heightened vaginal inflammation is associated with a non-optimal vaginal microbiota and confers increased risk of acquiring STIs such as HIV. As well as maintaining a low vaginal pH which restricts the growth of non-optimal bacteria, LA also has potent anti-inflammatory properties which may be beneficial *in vivo*. However, these beneficial effects may be undermined by cytotoxicity associated with delivery gel formulations. We therefore assessed the impact of vaginal OTC gels containing LA on the production of key pro- and anti-inflammatory factors by cervicovaginal epithelial cells. All hyperosmolal gels, including those containing LA, elicited substantial production of IL-1α and/or β similar to the Gynol II gel containing nonoxynol-9, and many also elicited increased MIP3α production (Figure 2). LA-containing gels, particularly those containing a high (>7%) LA content, were associated with lower IL-6 and IL-8 production, although this observation is confounded by their very high cellular toxicity shown in Figure 1 and the observation of lower IL-6 production from cells treated with both the placebo and Gynol II. Substantially enhanced production of IL-1RA was observed following treatment with all gels irrespective of LA content, with the positive control Gynol II (which also contains LA) also eliciting IL-1RA production. However, levels of this cytokine were above the limit of quantification of the assay so differences between individual gels could not be assessed.

Despite having minimal effect on cell viability or TEER, both iso-osmolal gels altered inflammatory factor production from epithelial cells. Compared to untreated tissues, Restore elicited increased production of IL-6 and IL-8 whilst Fleurstat elicited substantially increased production of IL-8 and MIP3α. These findings indicate that vaginal gels can induce substantial changes to the inflammatory status of cervicovaginal epithelial cells which may be both dependant and independent of cytotoxicity related to gel osmolality.

### Histological Effects of OTC Gels in the EpiVaginal Model

**Figure 3:**
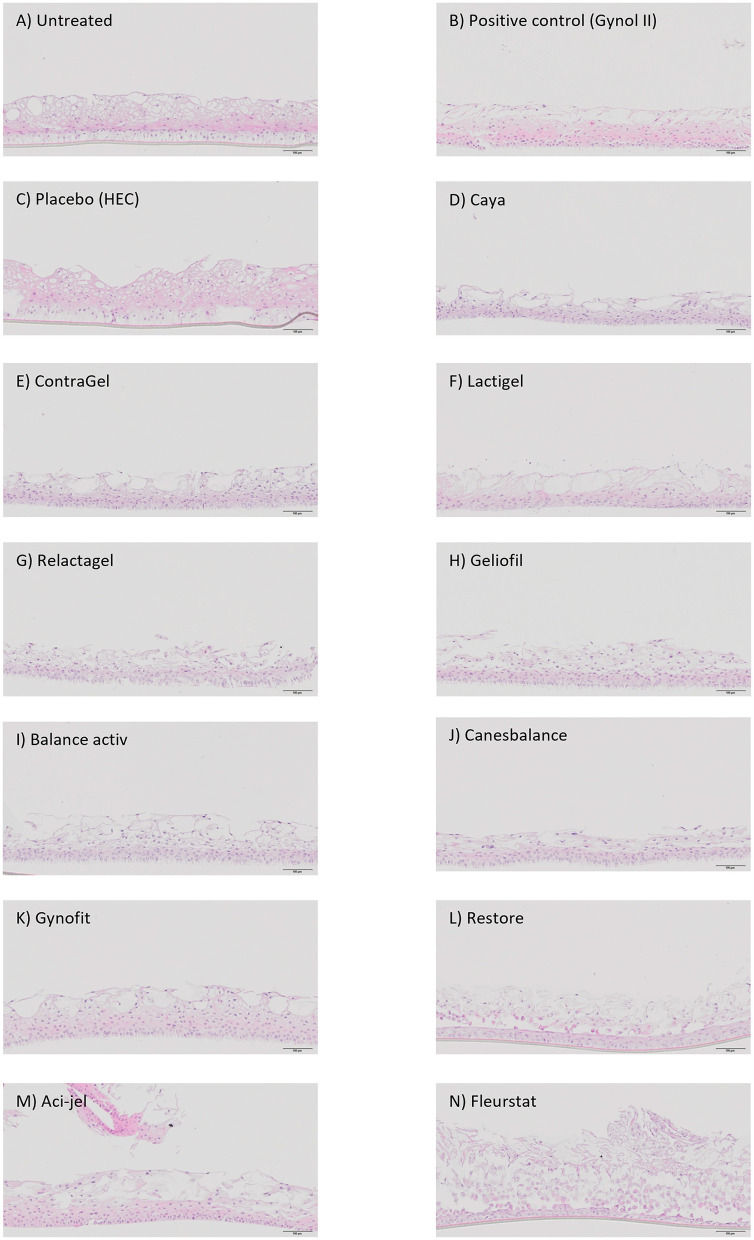
Histological analysis of EpiVaginal tissue. The impact of over-the-counter gels on the structure and morphology of the cervicovaginal epithelium was assessed microscopically after 24 h of treatment using hematoxylin and eosin staining. **(A-N)** show a representative image from each treatment.

As a qualitative measure to assess the effect of the various gels on epithelial structure, tissue samples underwent H&E staining and histological analysis (Table 2; Figure 3). Consistent with previous reports, the positive control gel Gynol II elicited severe tissue damage (indicated by damage to the basal and parabasal layers) compared to the untreated control whilst the placebo gel demonstrated no effect on tissue morphology. After 24 h of treatment, eight of the 11 OTC gels demonstrated moderate (++) to severe (+++) tissue damage while two exhibited mild damage and only one gel (Gynofit) had no significant morphological effect on tissue structure (Table 2). Disruption of epithelial structure was even observed following treatment with iso-osmolal gels, with Fleurstat treatment eliciting severe loss of glycogen filled layers and separation of disorganization of suprabasal, apical and glycogen layers.

**Table 2:** Summary of histological effects of over-the-counter gels on EpiVaginal tissue.

**Gel product**	**Loss of glycogen layers**	**Epithelial thinning**	**Epithelial layer separation**	**Epithelial reorganization**	**Tissue damage**
Untreated	-	-	-	-	-
Gynol II	++/+++	-	+++	-	+++
Placebo (HEC)	-	-	-	-	-
Caya Gel	-/++	-/++	-/++	-/++	+
ContraGel	+/++	+/++	+/++	+/++	++
Lactigel	-/+	+	++	-	++
Relactagel	++/+++	+/++	+	++/+++	++
Geliofil Classic	++	+	-	+	++
Balance Activ	+	++	++	++	++
Canesbalance	++	++	++	++	++
Gynofit	-	-	-	-	-
Restore	++	++/+++	++/+++	++	++
Aci-Jel Balance	-/+	-	-/+	-/+	+
Fleurstat	+++	++	+++	+++	++

*Histological examination assessed after 24 h of treatment. Table show results obtained from n = 2 independent assays. Where discrepancies were observed between assays both results are shown separated by “/”.-, no effect compared to negative control; +, minimal effect; ++, mild effect; +++, moderate/severe effect*.

## Discussion

A plethora of OTC gels are available for intravaginal use and whilst the active ingredients of these gels may possess desirable properties, the formulations in which they are delivered could counteract these benefits. Here, we confirm that while the *Lactobacillus* metabolite LA has been shown previously to elicit a range of beneficial properties *in vitro*, some of these effects may be masked by the considerable cellular toxicity elicited by hyperosmolal LA-containing gels and the production of inflammatory cytokines from cervicovaginal epithelial cells following treatment with both hyper- and iso-osmolal gels. Furthermore, the majority of OTC gels evaluated elicited gross alterations to the morphology and structure of the epithelium and abolished epithelial barrier integrity, which *in vivo* may have important implications for pathogen transmission through the vaginal mucosa.

Our intention in this study was to assess the safety of LA-containing OTC gels by evaluating their effects on cervicovaginal epithelial cell viability and barrier integrity as well as evaluate their immunomodulatory properties, given our previous findings regarding the ability of LA to inhibit pro-inflammatory responses *in vitro* (18, 19). However, interpretation of the soluble inflammatory factor data was confounded by high cytotoxicity of the hyperosmolal LA-containing gels. The finding that LA-containing OTC gels reduced production of pro-inflammatory factors IL-6 and IL-8 from epithelial cells is consistent with our previous findings with purified LA (18), but it remains possible that cell death may have affected the functionality of these cells. Indeed, heightened production of IL-1α and IL-1β was observed following treatment with hyperosmolal LA-containing gels but was also elicited by a gel containing Nonoxynol-9, which is consistent with the association of these factors with cellular toxicity (44). Our data are consistent with previous evaluations of vaginal lubricants in an alternative 3D vaginal epithelial cell model where hyperosmolality was significantly associated with cytotoxicity, morphological changes and altered production of inflammatory mediators (39). The detrimental impact of LA-containing gels evaluated here on epithelial integrity is likely a barrier for their recommended use in conditions such as BV where they may exacerbate mucosal abnormalities and elevate genital inflammation already present in women with BV. Therefore, further work is required to identify the optimal formulation for LA-containing gels to maximize desirable anti-inflammatory and bactericidal effects whilst mitigating off-target effects associated with cytotoxicity.

Whilst hyperosmolality clearly has a significant effect on epithelial cells (31, 39), even iso-osmolal gels were associated with some changes to inflammatory factor production, including substantially increased production of IL-6 and IL-8 which are part of a panel of inflammatory cytokines associated with heightened risk of HIV acquisition *in vivo* (23). Histological analyses also revealed structural changes to the vaginal epithelium elicited by iso-osmolal gels including epithelial thinning, reorganization and layer separation which were not indicated by TEER analysis alone, consistent with previous findings (31). The specific gel components responsible for eliciting these effects are not clear, but vaginal gels contain a variety of excipients and preservatives, as well as other organic acids including acetic acid (which itself has immunomodulatory properties) (19) that may act either alone or in combination with the effects of hyperosmolality. It is therefore imperative that formulations intended for *in vivo* use are thoroughly characterized in a relevant *ex vivo* model to adequately assess the combined effects of both active and inactive components.

Our data presented here and previous findings (31, 39) confirm that the majority of OTC vaginal gels available are hyperosmolal and elicit substantial disruption to the cervicovaginal epithelium *in vitro*. Effects on pro-inflammatory factor production as well as barrier function are therefore essential to evaluate in pre-clinical studies of vaginal gel prototypes but are not always included in pre-licensing safety evaluations. There is no ideal animal model for the human vaginal mucosa since humans are the only mammals with high levels of vaginal lactobacilli and a low vaginal pH (pH <4.5) (45). Traditionally, safety of vaginal products prior to clinical trials has been assessed using the *in vivo* rabbit vaginal irritation (RVI) test recommended by the United Stated Food and Drug Administration (USFDA). Whilst this model provides indications of systemic toxicity and gross histological endpoints, it fails to completely predict dangerous side effects in humans *in vivo*. A lack of appropriate models for assessing safety of vaginal products has been particularly problematic in the development of topical microbicides for HIV, where agents such as Nonoxynol-9 not only failed to protect against infection but were associated with damage to the epithelial mucosa and increased HIV acquisition risk (46, 47). The traditional RVI test alone failed to predict these damaging effects of Nonoxynol-9 on the epithelium, and the RVI model has been subsequently extended to include analysis of cellular activation and recruitment as well as production of inflammatory immune mediators in an attempt to overcome these limitations (44). Here, we assessed OTC gel using the EpiVaginal tissue model, which is a physiologically relevant human primary tissue model of the cervicovaginal epithelium which avoids animal welfare issues and is more sensitive than the RVI for identifying mucosal toxicity (37). Whilst physiologically relevant elements such as mucus and bacterial biofilms are not present, the MatTek model does recreate the 3-dimensional structure of the cervicovaginal epithelium including differentiated epithelial layers. We have previously shown this model is tolerant to apical treatment with up to 2% LA at pH 3.9 with minimal toxicity or effect on the epithelial barrier, confirming its suitability for evaluating the effects of agents such as the OTC gels described here with acidic pH (18). Using this model, we confirm gel composition plays a critical role in determining the overall effect of vaginal gels on the cervicovaginal mucosa and its inflammatory status and highlight the importance of undertaking preclinical evaluation of vaginal products using sensitive systems such as this to prevent unwanted *in vivo* side effects. The responsiveness of the EpiVaginal model to exogenous estrogen and progesterone (48) indicates hormones could also be added to the system described here to model the impact of OTC gels on the epithelium in reproductive age women.

Given their ability to inhibit the growth of BV-associated bacteria, lactobacilli and LA are promising agents for the treatment and prevention of BV. The LACTIN-V trial evaluated administration of a *Lactobacillus crispatus* probiotic after metronidazole treatment for BV and reported a significantly reduced rate of BV recurrence in the treatment arm as compared to those treated with metronidazole followed by a placebo (49). A systematic review of LA-containing vaginal products for treatment of BV conducted in 2019 identified a lack of quality evidence to support their clinical efficacy (29). However, a recent open label, randomized clinical trial of 7 days treatment with a gel containing 4.5% LA reported 47% of women experienced symptom resolution for BV 14 days post randomization (50). Whilst this was significantly inferior to the standard of care treatment with an antibiotic (metronidazole; 70% experienced symptom resolution at 14 days), LA-gel use was associated with fewer systemic side effects and was preferred by many women as compared to antibiotic treatment (51) despite the gel formulation being hyperosmolal, although endpoints regarding inflammation and barrier integrity were not assessed (52). The development of vaginal products containing lactobacilli and LA are therefore likely to be clinically useful, provided the potential issue of gel excipients eliciting inflammation and altering epithelial barrier integrity can be mitigated.

Vulvovaginal candidiasis (VVC) is a common infection in women; however in contrast to BV it is a fungal infection caused by *Candida* species (53). There are mixed reports that Candida utilizes or is inhibited by LA, which can be species and isolate dependent (54). A recent study found LA at low pH neither enhanced nor inhibited the growth of *Candida* spp. (55) while another study (56) reported that LA inhibits seven different *Candida* spp. with a minimal inhibitory concentration of 2.5 mg/ml (0.25%). Despite these mixed *in vitro* data, there is no clear evidence that lactic acid gels increase the risk of VVC as observed in clinical studies assessing the efficacy of LA gels for treatment of BV (29). Furthermore, a recent open label study that evaluated metronidazole compared to over-the-counter LA gels for the treatment of BV reported that in the 2 weeks after initiating treatment vaginal candidiasis was reported in 25 vs. 17% of women, respectively (50).

This study had some limitations; the impact of LA-containing OTC gels on cytokine production was confounded by the high toxicity of the currently available OTC gels. The use of MTT viability data alone to predict tissue associated cytotoxicity (such as skin) is a widely used and accepted assay method by the cosmetic industry to evaluate corrosion, irritation, and phototoxicity. However, additional mechanistic analyses to understand whether cytotoxicity mediated by the hyperosmolal LA containing gels was due to apoptotic or necrotic effects and if the soluble inflammatory markers correlated with cellular markers were not performed. Future evaluation of iso-osmolal, less toxic formulations at an earlier timepoint and with a larger number of experimental replicates than utilized in these preliminary evaluations is required to adequately define the immunomodulator properties of LA-containing gels.

Taken together, this study highlights that whilst LA-containing vaginal products have potential utility in enhancing the vaginal environment and promoting desirable sexual health outcomes, there is a need to develop formulations which more closely resemble the physiological environment of the female genital tract to preserve epithelial integrity and function.

## Data Availability Statement

The raw data supporting the conclusions of this article will be made available by the authors, without undue reservation.

## Ethics Statement

This study was reviewed and approved by the National Disease Research Interchange (NDRI, Philadelphia, PA) internal review board.

## Author Contributions

GT, ACH, LM, CSB, and JR were involved in study design and conceptualization. KG, SA, SC, and SEM contributed methodology. DT, KG, and SA generated experimental data. GT provided supervision and DT, KG, SA, ACH, and GT performed formal analysis and interpretation. ACH and GT wrote the manuscript. All authors reviewed and approved of the final version.

## Funding

GT was supported by the National Health and Medical Research Council (NHMRC) of Australia and NHMRC Senior Research Fellowship GNT1117748. This work was funded by the Australian Centre for HIV and Hepatitis Virology Research (ACH2) awarded to GT, ACH, CSB, SC, and SA, the MRFF Frontier in Health and Medical Research Stage One grant MRFF75913 awarded to GT, ACH, CSB, SC, SEM, JR, LM, and DHHS Victorian Health and Medical Research Project Grant awarded to GT.

## Conflict of Interest

KG and SA are employed by MatTek Corporation. GT and ACH are co-inventors on a patent pertaining to the immunomodulatory effects of LA. The remaining authors declare that the research was conducted in the absence of any commercial or financial relationships that could be construed as a potential conflict of interest.

## Publisher's Note

All claims expressed in this article are solely those of the authors and do not necessarily represent those of their affiliated organizations, or those of the publisher, the editors and the reviewers. Any product that may be evaluated in this article, or claim that may be made by its manufacturer, is not guaranteed or endorsed by the publisher.
